# Minimum toe clearance: probing the neural control of locomotion

**DOI:** 10.1038/s41598-017-02189-y

**Published:** 2017-05-15

**Authors:** Tim Killeen, Christopher S. Easthope, László Demkó, Linard Filli, Lilla Lőrincz, Michael Linnebank, Armin Curt, Björn Zörner, Marc Bolliger

**Affiliations:** 1 Spinal Cord Injury Center, University Hospital Balgrist, Forchstrasse 340, 8008 Zurich, Switzerland; 2 Department of Neurology, University Hospital Zurich, Frauenklinikstrasse 26, 8091 Zurich, Switzerland; 3 Department of Neurology, Helios-Klinik Hagen-Ambrock, Ambrocker Weg 60, 58091 Hagen, Germany

## Abstract

Minimum toe clearance (MTC) occurs during a highly dynamic phase of the gait cycle and is associated with the highest risk of unintentional contact with obstacles or the ground. Age, cognitive function, attention and visual feedback affect foot clearance but how these factors interact to influence MTC control is not fully understood. We measured MTC in 121 healthy individuals aged 20–80 under four treadmill walking conditions; normal walking, lower visual field restriction and two Stroop colour/word naming tasks of two difficulty levels. Competition for cognitive and attentional resources from the Stroop task resulted in significantly lower mean MTC in older adults, with the difficult Stroop task associated with a higher frequency of extremely low MTC values and subsequently an increased modelled probability of tripping in this group. While older adults responded to visual restriction by markedly skewing MTC distributions towards higher values, this condition was also associated with frequent, extremely low MTC values. We reveal task-specific, age-dependent patterns of MTC control in healthy adults. Age-related differences are most pronounced during heavy, distracting cognitive load. Analysis of critically-low MTC values during dual-task walking may have utility in the evaluation of locomotor control and fall risk in older adults and patients with motor control deficits.

## Introduction

Falls from standing height in adults over the age of 60 are associated with 1-year mortality as high as 33% and lead to considerable morbidity, reduced independence and financial burdens^1, 2^. Trips may account for over half of falls in the elderly^3^ and tripping over rugs or carpets alone resulted in 38000 adults over 65 being admitted to US emergency departments over 7 years^4^. Such trips during walking may result if insufficient clearance is maintained during swing phase to avoid uneven ground or unseen obstacles. The toe trajectory nadir that occurs at or very close to mid-swing, termed minimum toe clearance (MTC)^5–7^, is the gait event associated with the highest risk of unintentional ground contact^8, 9^.

Executive function and, more specifically, the ability to appropriately allocate attention to walking is increasingly regarded as crucial to gait control in healthy older adults, particularly under more challenging walking conditions^10–17^. Impairment of attentional control – the ability to appropriately allocate finite cognitive resources to information processing tasks^18^ – is associated with an increased risk of injurious falls in older people^19, 20^. The effect of additional cognitive load on MTC has been assessed both in overground and treadmill-based studies utilising various cognitive dual-task paradigms^5, 21–25^, which report conflicting effects on mean or median MTC (generally small decreases or no change). Variability of MTC also appears to be controlled with high priority during cognitive dual-task walking^21, 23^ despite significant increases in that of other gait parameters^26^.

Although also cognitively demanding, walking with restricted vision appears to be associated with minimal gait adaptations in healthy older adults^27^ and with a slightly increased mean MTC relative to normal walking^28–30^. This discrepancy may be due to attention being consciously diverted *towards* walking during a restricted vision task, rather than *away* from it, as when performing an unrelated cognitive task, resulting in tighter, conscious control of MTC. Relying solely on changes in MTC mean/median or variability values to understand tripping risk and/or locomotor control under challenging walking conditions implicitly assumes normal distribution of MTC values. However, this is rarely the case in groups or individuals^22, 29, 31^ and task-related shifts in frequency distribution may significantly increase an individual’s tripping risk. Such aspects have only been partially explored^8, 24, 31^.

Based on these ideas, we developed a paradigm to investigate the effect of cognitive load and attention on the control of MTC in healthy adults of all ages. The conditions used – visual restriction and cortical distraction by means of a modified Stroop task – are complimentary in that the former encourages the participant to consciously attend to walking to ameliorate a challenge they are aware of, while the latter greatly distracts attention from locomotion. We additionally assess MTC distribution and timing and perform probability modelling to indicate the risk of tripping under cognitive load and restricted vision. We hypothesise that condition effects on MTC will be most pronounced in the group of adults aged over 60 and that the characteristics of MTC frequency distributions will result in higher modelled tripping risk in this group.

## Methods

This two-centre study, carried out in accordance with the Declaration of Helsinki and Good Clinical Practice, was approved by the cantonal ethics committee of Zurich (KEK-2014/0004). Data were uploaded into a secure, tamper-proof clinical trials database (SecuTrial®, interActive Systems GmbH, Berlin, Germany). Healthy individuals were consecutively recruited via flyers and posters from the local area with a target of 20 males and 20 females in each of three, pre-defined age groups (20–39, 40–59, 60–80). All participants gave written, informed consent. Data collection was performed over two visits. In the first visit, participants underwent medical screening followed by a thorough neurological and orthopaedic examination and were excluded if any abnormality was detected, including colour-blindness. Upon inclusion, participants initially underwent 40 minutes of habituation on the treadmill during which they were familiarised with the test protocol. Subjects were blinded to the purpose of the study.

Participants returned 1–7 days later for gait analysis. The timed 25-foot walk test (T25FW) and the 10-metre walk test (10MWT) were performed simultaneously from a standing start in a hallway marked with both distances. The speed of the treadmill for all subsequent trails was set at 50% of maximal overground speed as a proxy for preferred treadmill velocity, defined as the mean velocity over two attempts at the T25FW. Motion capture (Vicon, Oxford, UK) was performed as participants walked normally on an instrumented treadmill (FDM-T, Zebris Medical GmbH, Germany) through which foot pressure data was also recorded at a sampling rate of 120 Hz. A modified Cleveland model^32^ (Motion Analysis Corp., Santa Rosa, CA, USA) reflective marker constellation was applied to the pelvis and lower limbs, with the great toe marker placed over the second metatarsal head. A standard Vicon Plug-in-Gait model was applied to the upper body^33^. Vicon Nexus 1.8.5 motion capture software was used to record three-dimensional, kinematic data at 200 Hz.

**Figure 1:**
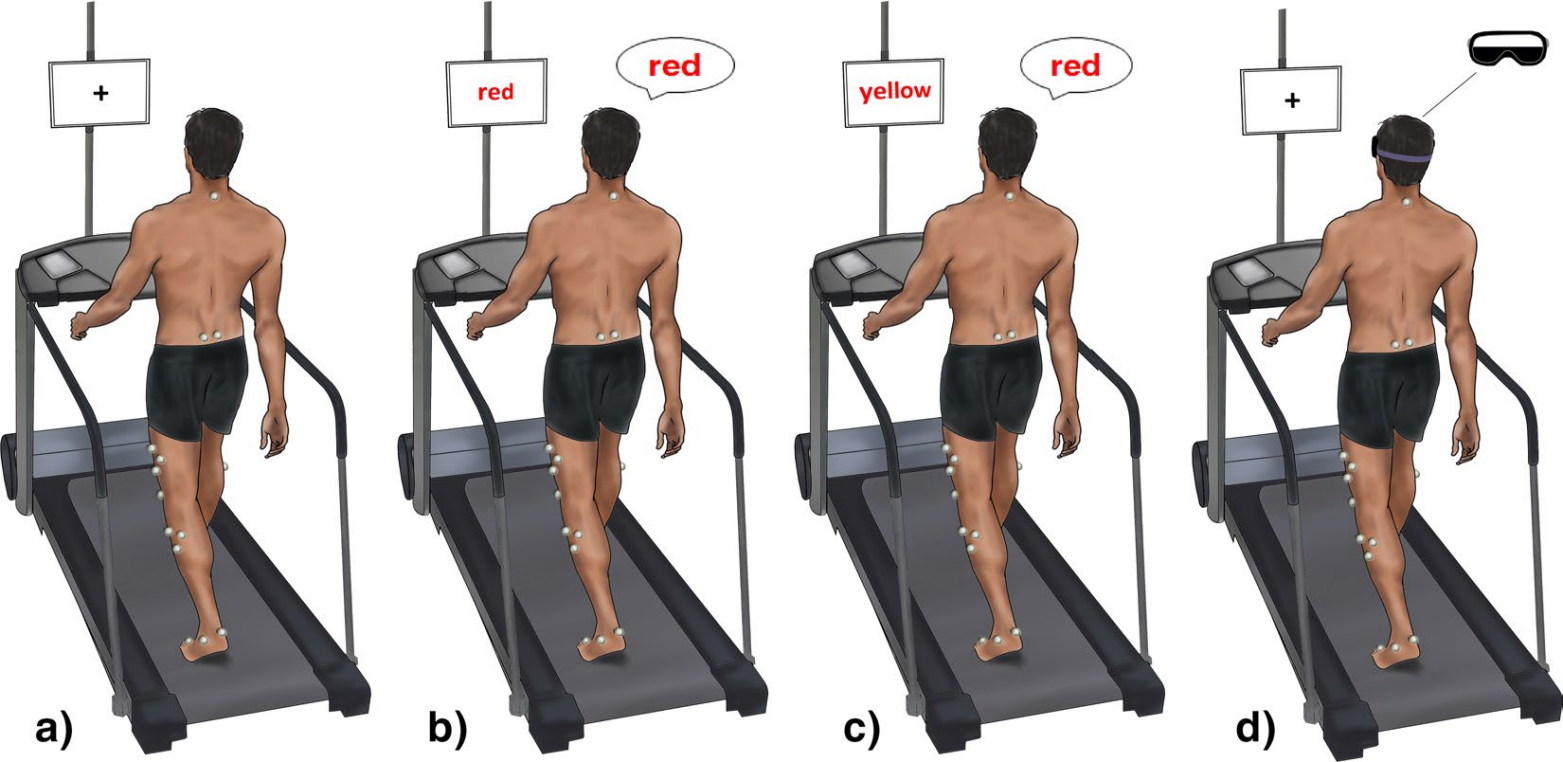
Experimental setup. Healthy adults aged 20–80 underwent 3D gait analysis while walking on an instrumented treadmill without handrail support. They undertook four locomotor tasks. Normal walking without a secondary task (**a**) was performed with the eyes fixed on a cross at eye height. Participants then walked while engaged in two Stroop colour-naming task (see methods) of differing difficulty. Image (**b**) shows the simpler task in which word and colour stimuli are congruent. In the more difficult, incongruent task (**c**) word and colour are discordant. Participants also carried out a visual restriction task in which they walked wearing eye goggles, the lower half of which were covered in black fabric to obscure the lower visual field. The upper edge of the fabric was affixed at the level of the subject’s interpupillary line. This figure was adapted from Fig. 1 in the publication Killeen *et al*. Increasing cognitive load attenuates right arm swing in healthy human walking. R. Soc. open sci. 2017 4 160993; DOI: 10.1098/rsos.160993. Published 25 January 2017 under the Creative Commons Attribution Licence 4.0.

Stable gait was recorded over 30–45 seconds as participants walked barefoot on the treadmill without handrail support. Participants were asked to walk under four different conditions. For the baseline, normal walking condition (NW), participants walked while fixing their gaze on a 22″ LCD monitor at eye height on which a cross was displayed (Fig. 1a). Two levels of additional cognitive loading were achieved by means of a modified Stroop word/colour naming exercise^34^ displayed on the same screen in place of the cross. In the first level (congruent Stroop; Fig. 1b), colour-words (red, blue, green or yellow), written in a colour consistent with the word, were presented at pseudorandom intervals in the participant’s self-declared native language and script. These intervals ranged between 600 and 1400 ms around a mean frequency of 1 Hz and the duration of a given stimulus was never within 200 ms of the one preceding it. This modification of the standard Stroop task^35^ was intended to avoid any entrainment of temporal gait parameters^36^ and to encourage constant attention, as the participant could not predict stimulus duration. In the second, more difficult level (incongruent Stroop; Fig. 1c), the word stimuli were presented in colours discordant with the written word. In both cases, participants aimed to state the colour in which the words were written as quickly and as accurately as possible.

In a fourth task, participants were asked to wear protective goggles which had been modified to obscure the lower half of the visual field (Fig. 1d). This was achieved by affixing black fabric to the lower half of the goggles with Velcro® at the level of the individual’s interpupillary line. Participants again fixed their gaze on the central cross. Trials were repeated if participants used the handrails or failed to maintain a safe position on the treadmill.

Marker trajectories were reconstructed, labelled, filtered and modelled in Nexus 1.8.5. A custom Matlab script (The MathWorks, Natick, MA, USA) was used to set gait cycle events from the synchronised treadmill force-plate data with foot-strike and foot-off defined by downward and upward 5 N threshold crossings respectively. Trials were manually inspected for recording and processing errors before per-stride spatiotemporal gait parameters were calculated using Procalc 1.1 (Vicon). Specifically, MTC was defined as the minimum difference in the vertical axis between the left or right great toe marker during swing phase and its trial minimum during stance.

The individual whose photograph was used as the basis of Fig. 1 was not a study participant and gave consent for the image to be published.

### Statistical analysis

Statistical analysis was performed using SPSS 24.0 (IBM Corp, Armonk NY, USA) and graphs produced using Prism 7.02 (Graphpad Software, La Jolla CA, USA). Attributes of the three age groups were compared using one-way ANOVA with post-hoc t-tests corrected for multiple comparisons with the Bonferroni method. The effect of locomotor condition on MTC and MTC timing, including the mean, median and coefficient of variation (CoV) of each parameter, were analysed using a linear mixed model in which condition (NW, congruent Stroop, incongruent Stroop, restricted vision) was a repeated measure. Fixed effects comprised condition, weight, height, age, gender and walking speed. Where significant condition effects were present, post-hoc t-tests were performed with Bonferroni correction and linear regression used to investigate relationships between scalar variables. Gait parameters of secondary interest, reflecting aspects of stability and gait control, were subjected to the same linear mixed model analysis. These comprised step width (+coefficient of variation; CoV), step length (+CoV) and the per-stride length of the 3D trajectory of the C7 marker and, to specifically assess mediolateral trunk sway, its 2D coronal component.

Analysis of MTC distributions was carried out by calculating the mean relative frequency of 1mm MTC bins for each age group. Each individual contributed MTC values for 25 strides to age group histograms. These data may also be presented as cumulative relative frequency plots by ordering all observations from smallest to largest. These distributions (i.e. 25 × *n* data points per histogram) were used for tripping probability modelling performed using a custom Matlab script based on the approach used by Best & Begg^8^, which takes into account skewness and kurtosis of the distributions to give the per stride probability of striking a hypothetical unseen object of a given size.

## Results

One hundred and fifty-seven individuals volunteered to take part in this study. Thirty-six were excluded at initial screening due to abnormalities of the neurological or musculoskeletal system. The most frequent reason for exclusion was prior surgery to the lower limbs or spine. One hundred and twenty-one participants completed the full protocol. All individuals completed the normal walking and congruent Stroop trials. Data was unusable in two of the incongruent Stroop trials (both in the middle-aged group), while three older individuals were unable to complete the visual restriction trial safely. The remaining 479 trials were available for analysis.

There were no significant group differences in mean height or weight or gender distribution but older adults walked more slowly in the T25FW (p ≤ 0.020) and 10MWT (p ≤ 0.010) and covered less distance in the 6-metre walk test (6MWT; p ≤ 0.031) than those in the younger age groups (Table 1). Accordingly, their mean fixed walking speed on the treadmill, set at 50% of the T25FW speed, was also somewhat slower (p ≤ 0.040). There were no significant differences between the younger and middle-aged groups.

**Table 1:** Demographics and walking ability in the three age groups.

Age group	n	Age (years)	Percent female	Weight (kg)	Height (cm)	Walking speed (m/s)	T25FW (s)	10MWT (s)	6MWT (m)
Young (20–39)	41	29.1 ± 5.0	51.2	70.1 ± 14.5	172 ± 8	1.15 ± 0.17	3.37 ± 0.45	4.41 ± 0.63	724 ± 74
Middle-aged (40–59)	40	47.7 ± 6.0	50	72.6 ± 15.5	172 ± 9	1.11 ± 0.14	3.49 ± 0.47	4.60 ± 0.59	710 ± 77
Older (60–80)	40	67.5 ± 6.0	47.5	68.6 ± 11.9	169 ± 8	**1.03 ± 0.16**	**3.79 ± 0.52**	**4.95 ± 0.73**	**664 ± 90**

Group comparisons were made using one-way ANOVA with post-hoc t-tests corrected for multiple comparisons (Bonferroni). Values given are means ± standard deviation. Bold type indicates significant difference compared to both other age groups at the p ≤ 0.05 level. T25FW; timed 25-foot walk, 10MWT; 10-metre walk test, 6MWT; 6-minute walk test.

**Figure 2:**
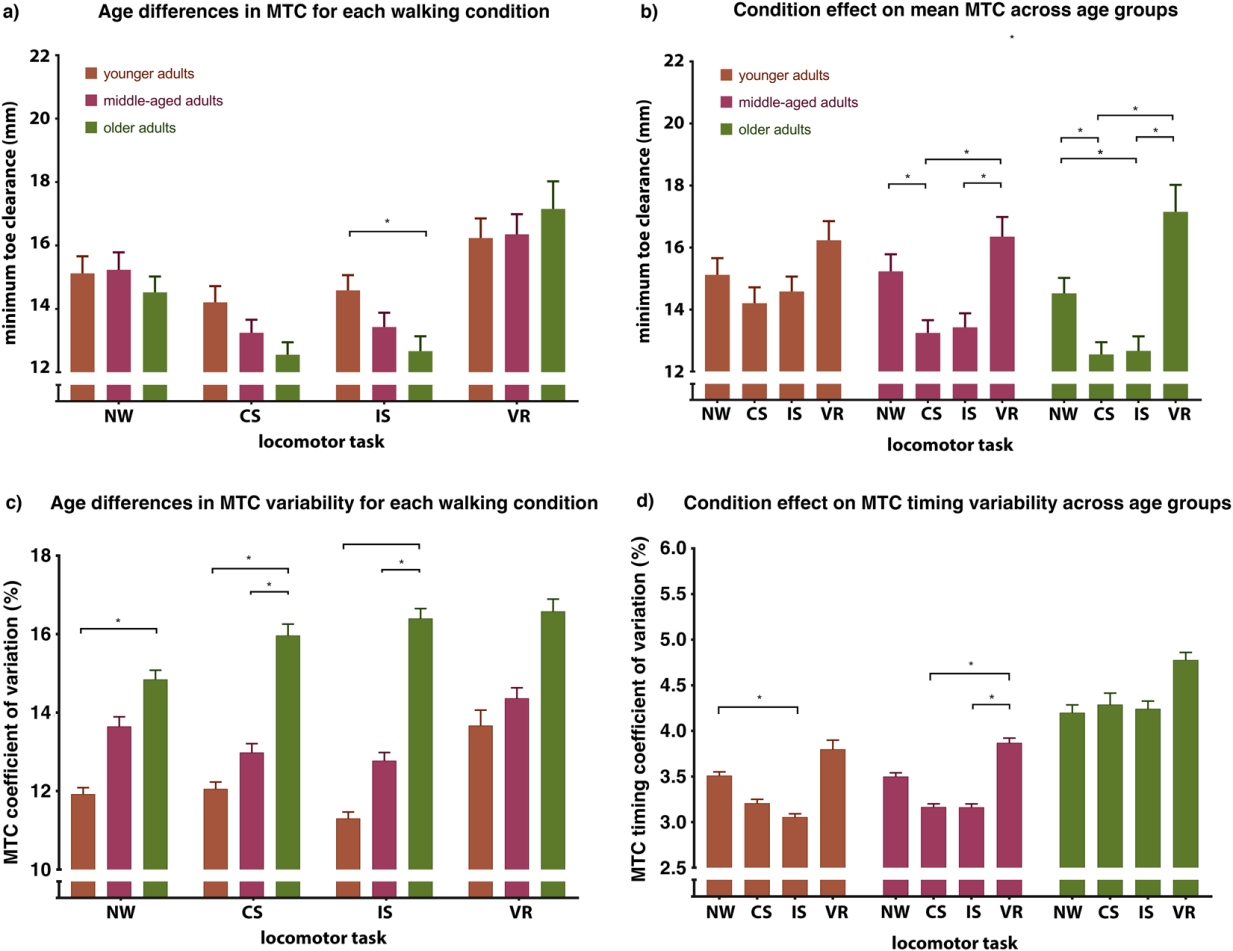
Minimum toe clearance parameters under different locomotor conditions. (**a**) The effect of age group on MTC in each of the four walking conditions. Differences in mean MTC between age groups (younger adults; 20–39, middle-aged adults; 40–59, older adults; 60–80) tested using ANOVA and post-hoc t-tests where appropriate with significance set at p ≤ 0.05, corrected for multiple comparisons (Bonferroni). NW; normal walking, CS; congruent Stroop task, IS; incongruent Stroop task, VR; visual restriction. (**b**) Within-age group condition effects on mean MTC, compared using a linear mixed model (see methods) and post-hoc t-tests where appropriate with significance set at p ≤ 0.05, corrected for multiple comparisons (Bonferroni). (**c**) Differences in mean MTC variability (coefficient of variation; CoV) between age groups, compared using ANOVA as in (a). (**d**) Condition effect on MTC timing variability (CoV), compared using a linear mixed model as in b). Error bars indicate SEM.

**Figure 3:**
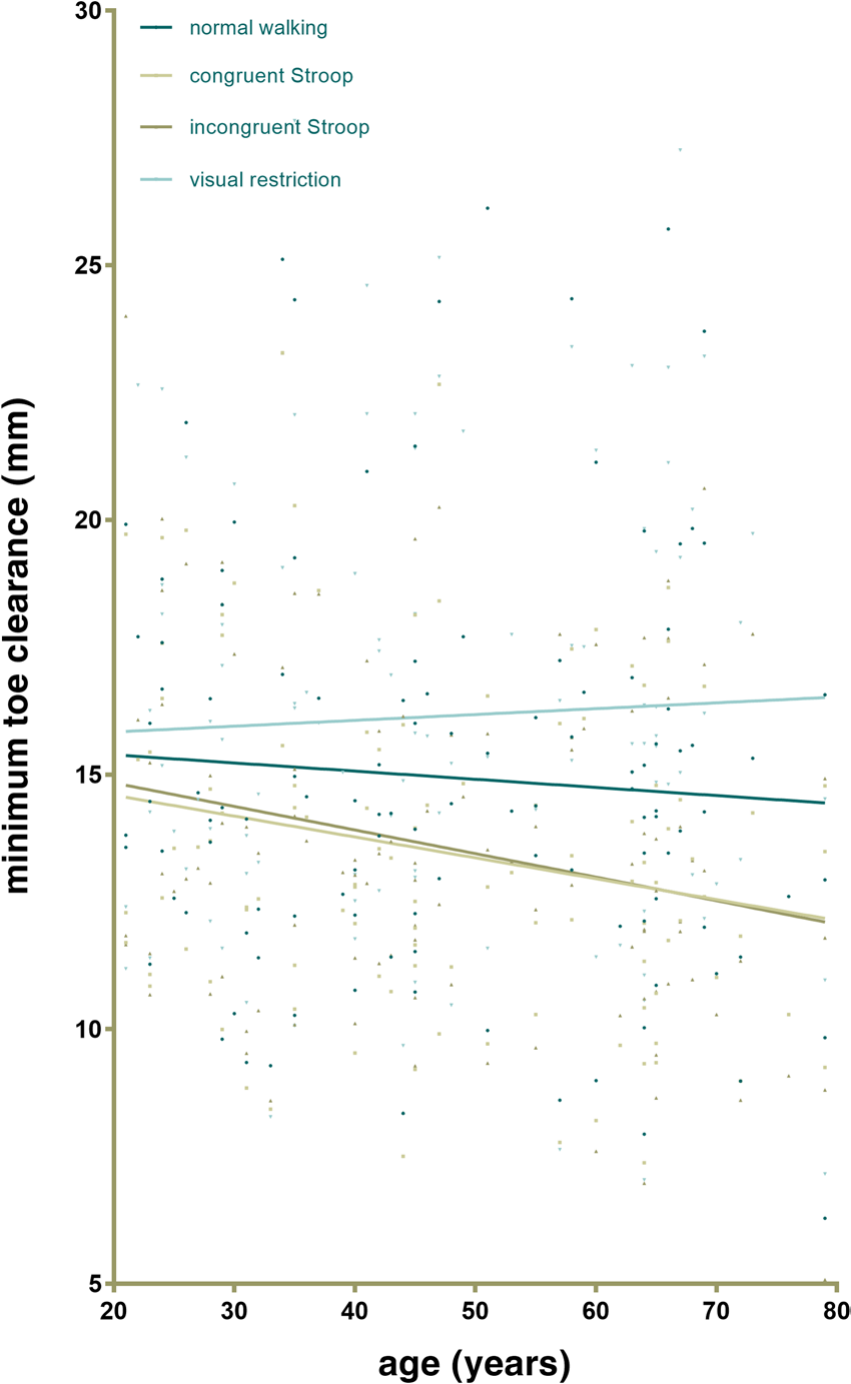
Scatter plot of age and mean minimum toe clearance under four walking conditions. During the congruent and incongruent Stroop tasks, age was a significant, but weak, negative predictor of MTC, with R^2^ values of 0.043 (F = 4.9; p = 0.030) in the congruent and 0.050 (F = 6.0; p = 0.016) in the incongruent task. During normal walking and visual restriction, no such relationship was observed.

During normal treadmill walking, mean MTC was 15.0 mm (median 14.5 mm) with a standard deviation of 4.0 mm (interquartile range 2.1 mm) for the whole cohort. There was no difference between the age groups in terms of MTC during normal walking, with values (mean ± SEM) of 15.1 ± 0.5 mm, 15.2 ± 0.6 mm and 14.5 ± 0.5 mm in the younger, middle-aged and older groups, respectively (Fig. 2a). However, age differences did begin to become apparent during the cognitive dual-tasks, with older adults demonstrating significantly smaller MTC values than those aged 20–39 during the incongruent Stroop task (12.7 ± 0.5 mm *vs* 14.6 ± 0.5 mm; one-way ANOVA with post-hoc t-tests, p = 0.020). There were no significant differences in MTC values across age groups during the visual restriction task. During both cognitive tasks, age was a significant, but weak, negative predictor of MTC, with R^2^ values of 0.043 (F = 4.9; p = 0.030) in the congruent and 0.050 (F = 6.0; p = 0.016) in the incongruent Stroop tasks (Fig. 3). During normal walking and visual restriction, no such relationship was observed.

Due to positively skewed distributions of most MTC histograms, median MTC values were generally marginally lower than the means but followed the same pattern with respect to condition-related changes. For completeness, these values are displayed in Supplementary Figure 1.

### Condition effects on MTC and associated parameters

The trial minimum of the ipsilateral toe marker was used as the ground reference for determining MTC. Over all trials, the standard deviation of this value with respect to position of this marker relative to the treadmill frame during normal walking was 1.12 mm in the vertical axis. This variability was not significantly different across age groups or walking conditions.

Locomotor condition (p < 0.001) was among four parameters revealing significant main effects on MTC within the linear mixed model. The others were gender (p = 0.037), weight (p = 0.004) and walking speed (p = 0.000). Post-hoc linear regression during normal walking revealed that walking speed was a weak, yet significant, positive predictor of MTC (R^2^ 0.121; F = 17.1; p = 0.000).

Corrected, post-hoc comparisons revealed that, under the congruent Stroop task, MTC decreased significantly relative to normal walking (Fig. 2b) in the middle-aged (13.2 ± 0.4 mm; p = 0.030) and older groups (12.6 ± 0.4 mm; p = 0.016). A similar reduction in MTC was seen during the incongruent Stroop in the older age group (12.7 ± 0.5 mm; p = 0.050). Generally, visual restriction was associated with a rebound of MTC to mean values similar to those of normal walking, with MTC during visual restriction significantly higher than that in both cognitive tasks in middle-aged (16.4 ± 0.6 mm; p ≤ 0.002) and older adults (17.2 ± 0.9 mm; p ≤ 0.004).

In older adults, MTC CoV was significantly higher compared to that of younger adults in all tasks except visual restriction (ANOVA with post-hoc t-tests; p ≤ 0.032, Fig. 2c). No group showed any significant task-related changes in overall MTC CoV.

Absolute timing of MTC did not vary significantly between age groups or under the different walking conditions within age groups (data not shown), with MTC occurring at 57 ± 3.6% (±SD) of swing phase in the overall cohort. Stride-to-stride variability of this metric did show some significant changes, with MTC timing coefficient of variation (CoV) reduced in younger adults during the incongruent Stroop task (mean ± SEM; 3.51 ± 0.13% vs 3.06 ± 0.11%; p = 0.049). In the middle-aged group, MTC timing CoV increased with visual restriction (3.87 ± 0.17%) relative to both Stroop tasks (congruent; 3.17 ± 0.11%; p = 0.02, incongruent; 3.16 ± 0.12; p = 0.002, Fig. 2d). Older adults showed no significant condition effect, but had significantly greater MTC timing variability compared to both younger groups in all locomotor tasks (data in Fig. 2d, p ≤ 0.04; comparisons not shown).

No task-related changes were seen in step width, step width CoV, step length or step length CoV in any group (Supplementary Table 1). However, trunk stability was impaired in the older age group under cognitive load, with the 3D trajectory of the C7 marker significantly increased (congruent; 186.7 mm, incongruent; 190.2 mm) relative to normal walking (164.0 mm).

### Condition effects on MTC frequency distributions

**Figure 4:**
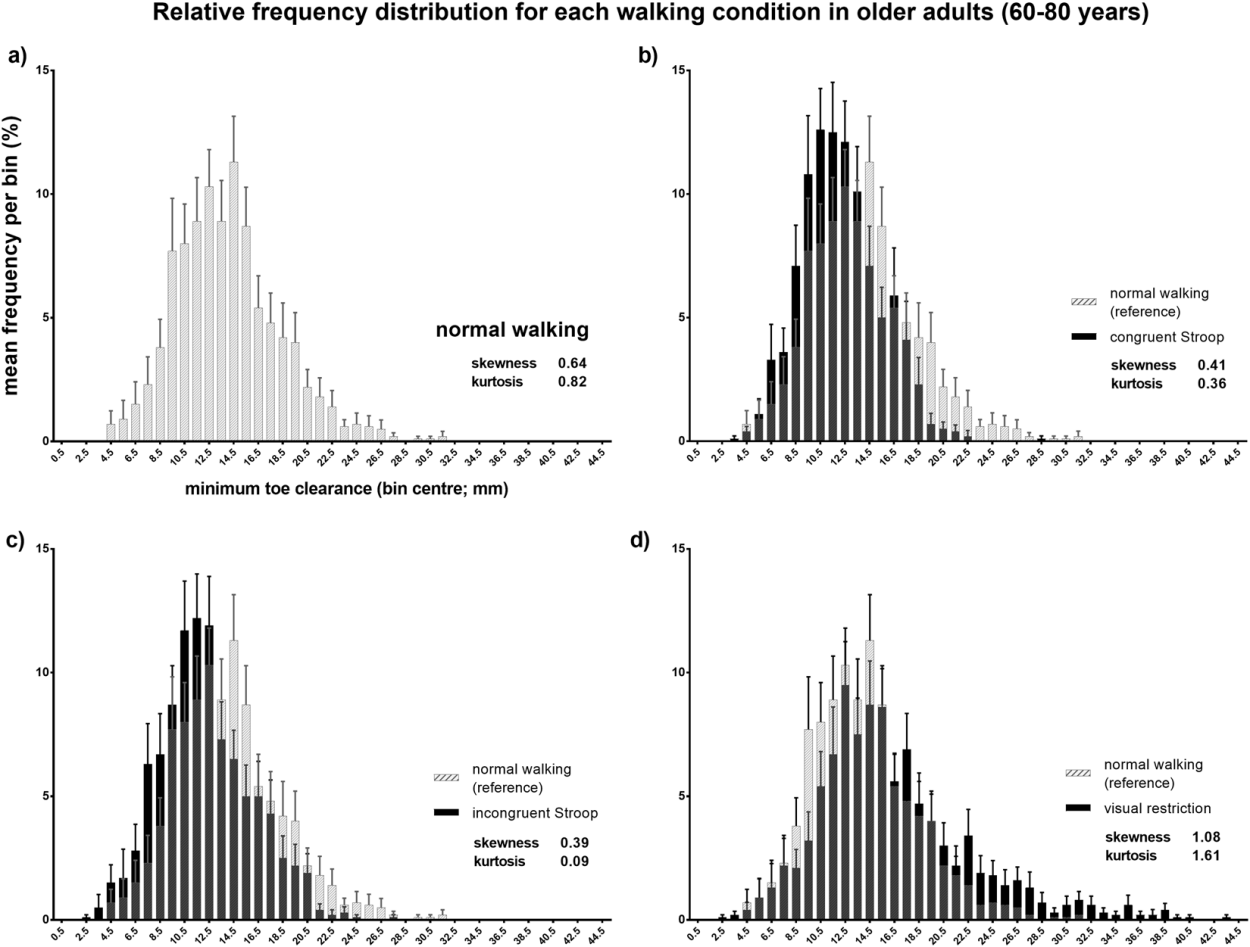
Relative MTC frequency distributions for healthy adults aged 60–80 years. Each individual contributed MTC values for 25 consecutive strides. Values indicated are mean frequencies per 1 mm bin with error bars indicating standard error of the mean. The histogram for normal walking is indicated in (**a**) and is presented as a semi-transparent overlay (grey) to allow comparison with the histograms of the three locomotor conditions (black; (**b**–**d)**). Similar graphics for the younger and middle-aged cohorts may be found in the Supplementary Material.

**Figure 5:**
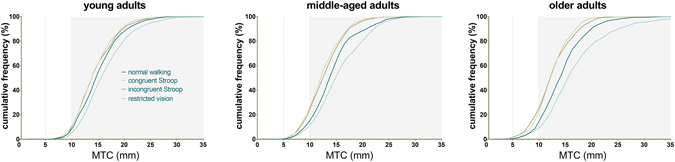
Minimum toe clearance cumulative relative frequency graphs for each age group. Each individual contributed 25 consecutive MTC values to the group histogram. Dotted lines indicate MTC thresholds of 5 mm and 10 mm, while shaded area indicates MTC values over 10 mm. MTC; minimum toe clearance.

None of the group MTC histograms were normally distributed, with all demonstrating positive skewness and all but one (visual restriction in adults aged 40–59) were leptokurtic (Fig. 4 and Supplementary Figure 2). Visual restriction resulted in a histogram shifted towards higher MTC values, with marked increases in skewness. Conversely, lower MTC values and decreased kurtosis and skewness were associated with the Stroop tasks. Cumulative relative frequency plots of the four walking conditions for each of the three age groups are displayed in Fig. 5. While in younger and middle-aged adults there was little difference in the distribution of MTC values under the two levels of the Stroop task, in older adults a dissociation was observed, with the more demanding, incongruent Stroop task associated with notably higher frequencies of extremely low MTC values (below 10 mm; Fig. 5).

**Figure 6:**
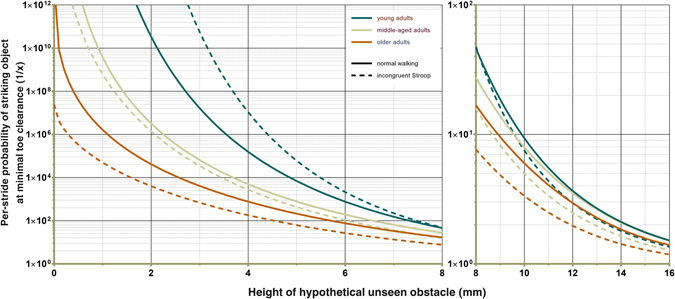
Tripping probability modelling for healthy adults aged 20–80 years during normal walking and under increased cognitive load. Modelling was based on the group frequency distributions and followed the approach taken by Best and Begg^8^. Briefly, per-stride probabilities of striking a hypothetical, unseen obstacle of a given height at MTC are modelled based on MTC frequency distributions, including skewness and kurtosis. Similar graphics for all conditions and age groups may be found in the Supplementary Material.

Tripping probability modelling yielded curves derived from each group histogram (Fig. 6 and Supplementary Figure 2a,b). In older adults, all walking conditions were associated with a higher risk of tripping over hypothetical, unseen objects of all heights compared to that of the two younger groups (Supplementary Figure 3). Both Stroop tasks were associated with elevated tripping risk relative to normal walking in all ages. In the older age group, the dissociation of the frequency distributions at extremely low MTC values under the different levels of cognitive distraction manifests as markedly higher tripping risk during the more demanding of the two Stroop tasks below 10 mm (Fig. 6). Data for one individual was removed from the younger age group for the calculation as inclusion of this data caused the probability modelling for the incongruent Stroop task to fail, likely because the distribution pattern was biphasic.

## Discussion

The search for unequivocal gait markers of tripping risk in older adults and patients with impaired locomotion has been frustrated by high inter- and intra-individual variability of candidate parameters or low specificity^37, 38^. Here we demonstrate that a combined analysis of the response of MTC to different locomotor conditions, including distribution analysis, is feasible and capable of revealing age-dependent and task-specific differences in motor control mechanisms in a large cohort of healthy adults.

MTC is predominantly mediated by dorsiflexion at the ankle joint around mid-swing^39^ and it is thought that tibialis anterior is under more direct corticospinal control than the extensors active in early swing phase^40–42^. Thus, it is reasonable to anticipate kinematic adaptations to changes in the degree of supraspinal control of walking to manifest in attributes of MTC. The results presented here are compatible with a simple hierarchical model of motor control^43, 44^, in which depletion of attentional resources by the Stroop task causes a shift towards a more autonomous, self-organised gait pattern characterised by reduced MTC, a less skewed MTC distribution and perhaps reduced variability^44^. On the other hand, restricting vision encourages the effortful intervention of higher levels of the CNS, resulting in kinematic adaptations aimed at reducing the likelihood of floor contact.

At 15.0 mm, the mean MTC during normal treadmill walking in this study was similar to central tendency values at preferred speed reported previously (14.9–15.6 mm)^6, 22, 31^. Also in line with previous work, no significant differences in baseline (i.e. normal walking) MTC were observed between age groups^22^. There is no ideal measure of central tendency for invariably non-normally distributed MTC data. We chose the mean as our main descriptor for two reasons: the number of gait cycles (25) was relatively low, meaning the median is susceptible to sampling fluctuations and the mean is reported more frequently in the MTC literature, allowing comparisons. Due to the positive skewing of nearly all histograms, median MTC was nearly always somewhat smaller than the corresponding mean. We report the median values for the main comparisons in Supplementary Figure 1.

As expected, condition effects were most pronounced in the healthy elderly, with significant decreases in MTC under cognitive load and a trend towards elevated values under visual restriction. These results corroborate both the tendency towards small MTC decreases observed during diverse dual-task experiments (answering questions^45^, serial subtractions^21^) and MTC increases during visual blurring or restriction^29, 46^. In keeping with earlier research, MTC CoV during normal walking showed a significant increase with age^22, 31^ yet was not significantly modified by cognitive loading^21^ or visual restriction^28^. This suggests that a strategy of reducing overall MTC variability to minimise critically low MTC values described elsewhere^23^ is not, in fact, utilised by healthy adults under increased cognitive load. Other spatiotemporal parameters were remarkably insensitive to the different conditions, although a significant increase in upper body sway was observed during the Stroop tasks in the elderly. This may be related to postural instability, an artefact of articulation during the task^18^ or may be related to increased arm swing asymmetry, which is known to result from engaging in the Stroop task^47^.

Aging is associated with recruitment of a broader range of brain structures during gait control compared to younger adults, particularly the prefrontal and basal ganglia networks^39, 40^ that also represent the neural substrate of Stroop task performance^11, 38, 41, 42^. When attentional resources are consumed by the relatively mild cognitive demands of the congruent Stroop task, the elderly CNS produces a narrower range of MTC values. While overall CoV is barely affected, a marked deskewing away from higher MTC values is seen (Fig. 4b), and kurtosis decreases towards 0, i.e. a more normal distribution. This is possibly an attempted safety strategy utilising preserved peripheral visual cues^30, 31^, although it is difficult to see the utility of eliminating high outlier MTC values with the small yet significant drop in mean MTC and the attendant increase in tripping risk. Instead, this move towards a suboptimal, normal distribution are most compatible with a switch to a more stereotyped, automated motor control strategy dominated by the brainstem and spinal cord as higher attentional resources are directed to the Stroop task^26^. During the *congruent* Stroop task, older adults maintain enough attentional control to minimise extremely low, dangerous MTC values. When cognitive load is increased further in the more difficult, *incongruent* task, however, supraspinal processing of visual and sensory afferent information competes for limited attentional resources and the influence of the brainstem and spinal cord systems on the locomotor pattern increase at the expense of higher levels of control^44^. In younger adults, this lower-order system is capable of producing safe MTC parameters^23^. During healthy aging, however, this mechanism may gradually become less reliable due to a switch towards the prioritisation of balance^48, 49^, degradation of the afferent pathways on which rhythmic spinal centres depend^50^ or a general deterioration in gait automatism in old age^15, 44, 51^, to the degree that potentially critical MTC events occur (Fig. 4c).

Visual restriction, in which attentional resources are freely available yet feedback is degraded, results in a converse strategy that is imperfectly implemented by older adults; the locomotor system compensates by amplifying skewness towards higher MTC values (Fig. 4d). In contrast to the more automatic pattern under cognitive load, walking without visual feedback results in highly skewed MTC distributions in keeping with cautious, tight control of MTC reliant on the other senses available to the CNS^28, 52^. Unlike cognitive distraction, participants were keenly aware that their locomotor system was being challenged and likely switched conscious attention to control of MTC. Perhaps once more due to impaired proprioception and/or descending motor control in older adults, the frequency of extremely low MTC values increases despite this strategy of tighter control and tripping risk rises.

While changes in mean MTC and variability in all tasks were small in absolute terms, probability modelling of the group histograms show that the changes in MTC distribution characteristics brought about by secondary tasks can have substantial effects on the theoretical risk of tripping. MTC values ≤10 mm at the left of the distribution curve have profound consequences. For an unseen, 4mm obstacle conflicting with MTC, the per-stride risk of tripping for a young adult walking normally with no dual-task is approximately 1:15000, while for a healthy, older individual engaged in a demanding cognitive task, the same scenario is associated with a 1:180 risk of contact (Fig. 6). It is also possible to use the same approach to model the MTC variability distributions associated with those of the mean values and then to test for significant differences at given hypothetical obstacle heights. However, to perform meaningful comparisons of these modelled probabilities, studies including cohorts of patients and elderly individuals with a history of falls would be required to determine values that indicate increased fall-risk.

In our cohort, mean MTC timing was entirely resistant to dual-task effects, confirming the findings of Santhiranayagam *et al*.^23^. Interestingly, variability of MTC timing was the only parameter to undergo any significant adaptation in healthy young adults, with a significant reduction seen during the more demanding, incongruent Stroop task. This effect lost significance in the middle-aged cohort and disappeared entirely in healthy older adults, in whom MTC timing was high relative to young adults across all tasks (Fig. 2d). These are unexpected results, as cognitive/locomotor dual-task effects are usually more pronounced in older adults^26^. They are also in contrast to findings in the spatial MTC variability domain in this study (Fig. 2c), in which MTC height variability was unaffected by walking task, and imply different control mechanisms^53^. Temporal aspects of gait, including MTC timing, may be more readily delegated to subcortical, brainstem and spinal locomotor components in the event of attentional resources being reallocated to a secondary task^26, 54^, resulting in more constrained MTC timing. As MTC timing is considerably less critical to tripping risk than toe clearance itself, this trade-off is beneficial in a dual-task setting.

There is large heterogeneity in the design and conduct of cognitive dual-task paradigms in gait analysis^26^. Most approaches include rhythmic stimuli and/or verbal responses which may entrain cadence or other spatiotemporal gait parameters and confound condition effects^36, 55, 56^. Furthermore, anticipation of intervals between stimuli allows participants the opportunity to revert their attention and cognitive resources to walking, resulting in a fluctuating and unpredictable degree of cognitive load. The modified Stroop task employed here aims to ameliorate these issues and provide a constant level of attentional and cognitive distraction. In this study, treadmill walking speed was set at 50% of each individual’s maximal overground walking speed. We employ this objective speed-selection approach in the clinical setting, as it allows us to challenge participants with dissimilar walking abilities to a proportional degree, irrespective of the many factors which may influence preferred treadmill speed^34, 57^. Healthy participants of all ages and body types thus walked at a speed that was proportional to their walking ability and which all perceived as comfortable. The small yet significant difference in absolute walking speeds between age groups (Table 1) may potentially influence MTC; indeed, higher walking speeds are associated with increased MTC values^39, 58^. In an overground study of unilateral transtibial amputees, intra-individual increases in walking speed from 0.97 to 1.36 m/s resulted in a 2.9 mm increase in MTC^39^. We believe such an inter-group effect of walking speed to be minimal in our sample, as the absolute difference in speed was small (0.14 m/s) and there were no significant differences in MTC between age groups during normal walking.

This study used group histograms to characterise MTC distribution and model tripping risk based on an established approach^8^. Caution should be exercised in interpreting the results of the probability modelling as the approach assumes that the hypothetical object will remain unseen and that it passes under the foot at MTC and not at any other point of swing phase. These “risks” should thus be seen as relative to one another and not indicative of absolute likelihoods, which are subject to myriad factors. Importantly, we did not consider heel clearance which is usually closer to the ground than the toe during the last third of the swing phase^59^. As heel height reaches zero at heel-strike, determining a meaningful heel clearance parameter that relates to tripping (i.e slipping) risk in this phase is difficult and beyond the scope of this paper. Our main aim was to provide norm data against which patients with neurological injury and disease may be compared during clinical, treadmill-based gait analysis. Translating these findings to overground walking should be done with caution as variability parameters are known to differ significantly between treadmill and overground walking^60^. Overground MTCs may generally be smaller^45^ and MTC increases under lower visual field restriction may be more marked on the treadmill due to the absence of optic flow or the view of the path ahead as additional compensatory cues^29^. While we used a sophisticated gait analysis system, MTC is a relatively simple parameter to calculate and can be measured using affordable systems in a clinical setting^61^. Future work should concentrate on characterising MTC values under dual-task conditions in patients and older adults known to be at risk of falling. This should include analysis of distribution and the frequency of extremely low MTC values. Such an approach may yield sensitive and specific gait biomarkers for neurological walking disorders, specific lesions and fall risk.

Here we provide comprehensive toe clearance data for adults free of orthopaedic or neurological disease walking on a treadmill. The findings suggest that the analysis of low outlier MTC values, rather than mean MTC, during dual-task treadmill walking may be a useful indicator of motor control ability, including fall risk, in older adults. Application of this approach to other populations with impaired motor control, such as patients with brain and spinal cord lesions, may also prove to be a sensitive gait biomarker for rehabilitation and other treatments designed to improve locomotor control in these groups.

## Electronic supplementary material

Supplementary Figures
