# Supplementary material for: Minimum toe clearance: probing the neural control of locomotion
Source: Sci Rep. 2017 May 15;7:1922. doi: 10.1038/s41598-017-02189-y (PMC5432520; doi:10.1038/s41598-017-02189-y)
Supplement: Supplementary file 1 — Supplementary Figures [file 41598_2017_2189_MOESM1_ESM.pdf]

Minimum toe clearance: probing the neural control of locomotion

Tim Killeen,[1*] Christopher S Easthope,[1] László Demkó,[1] Linard Filli,[2] Lilla Lőrincz,[2] Michael Linnebank,[3] Armin Curt,[1] Björn Zörner,[1†] Marc Bolliger[1†]

Supplementary Material

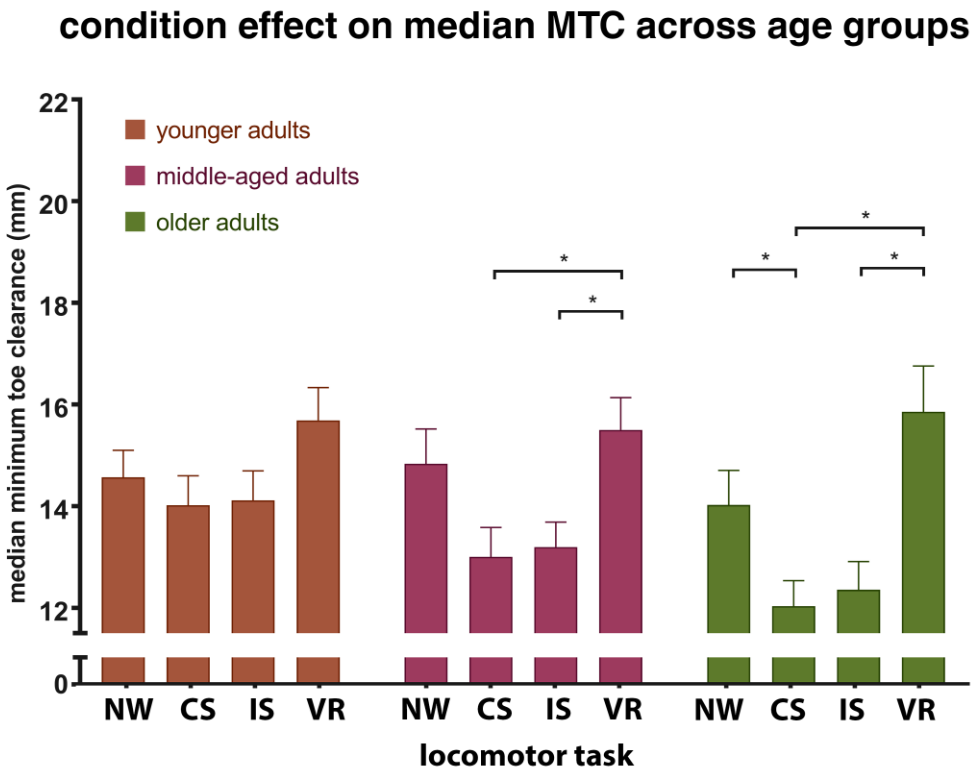

Supplementary Figure 1. Median minimum toe clearance under different locomotor conditions.

Within-age group condition effects on median MTC, compared using a linear mixed model (see methods) and post-hoc t-tests where appropriate with significance set at $p \leq 0.05$, corrected for multiple comparisons (Bonferroni). Error bars indicate SEM. NW; normal walking, CS; congruent Stroop task, IS; incongruent Stroop task, VR; visual restriction.

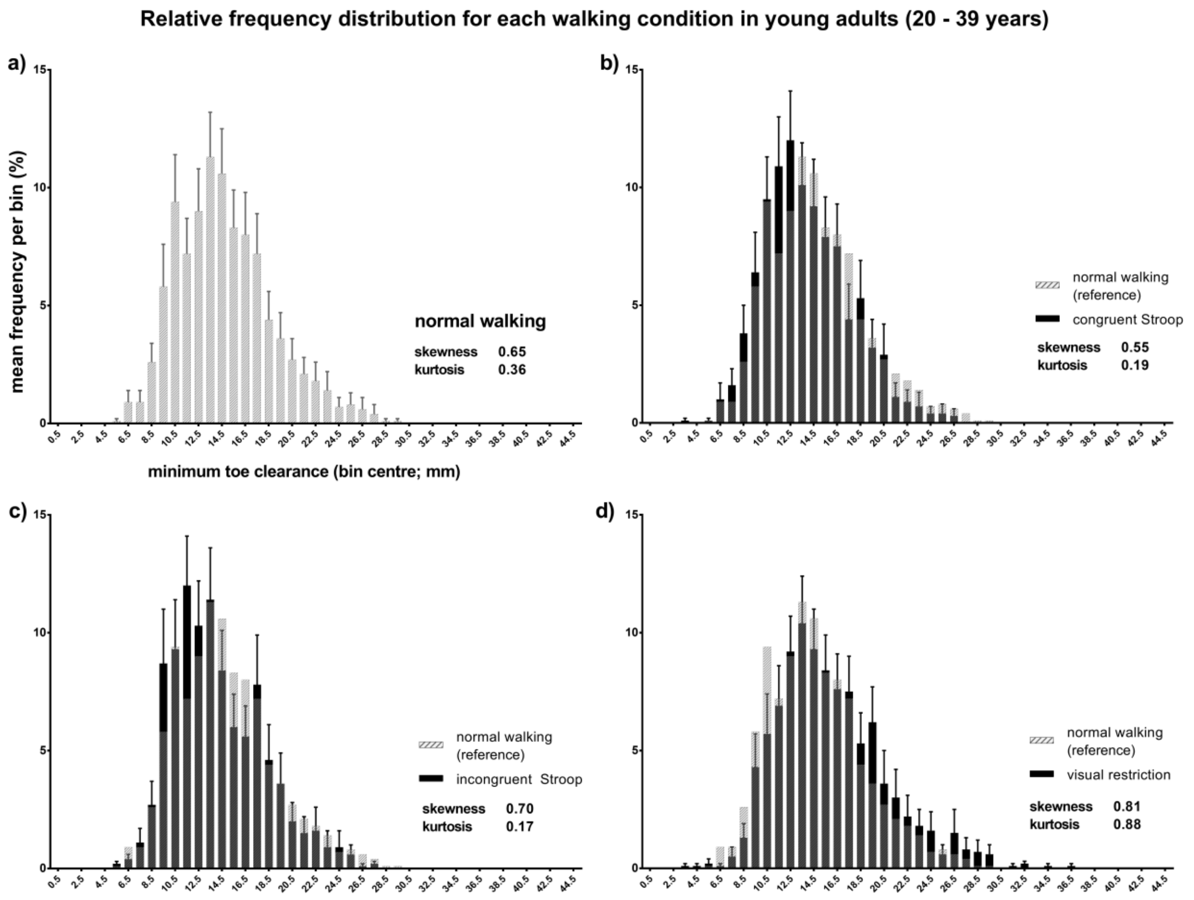

Supplementary Figure 2a. Relative MTC frequency distributions for healthy adults aged 20-39 years. Each individual contributed MTC values for 25 consecutive strides to the group histogram. Mean values are given as mean frequencies per 1mm bin with error bars indicating SEM. The histogram for normal walking is indicated in (a) and is presented as a semi-transparent overlay (grey) to allow comparison with the histograms of the three locomotor dual tasks (black; b-d).

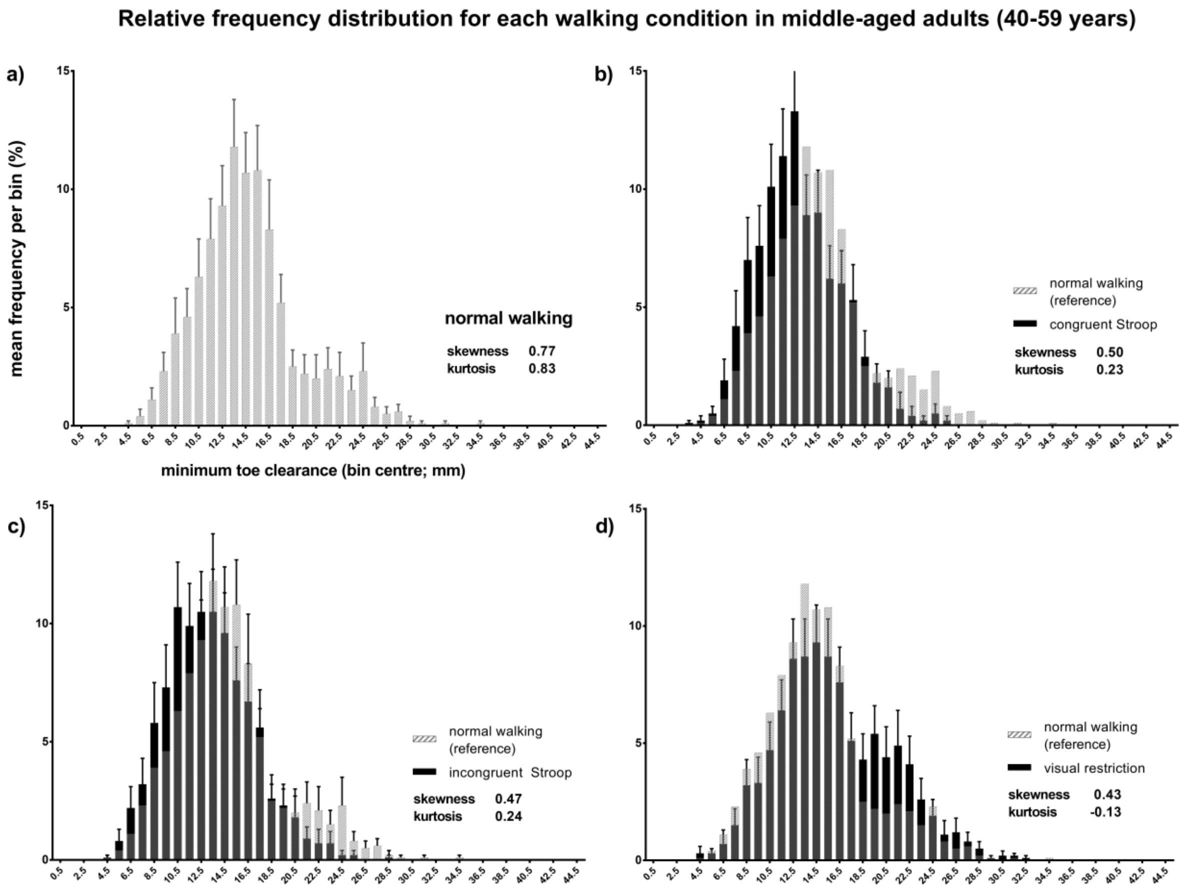

Supplementary Figure 2b. Relative MTC frequency distributions for healthy adults aged 40-59 years. Each individual contributed MTC values for 25 consecutive strides to the group histogram. Mean values are given as mean frequencies per 1mm bin with error bars indicating SEM. The histogram for normal walking is indicated in (a) and is presented as a semi-transparent overlay (grey) to allow comparison with the histograms of the three locomotor dual tasks (black; b-d).

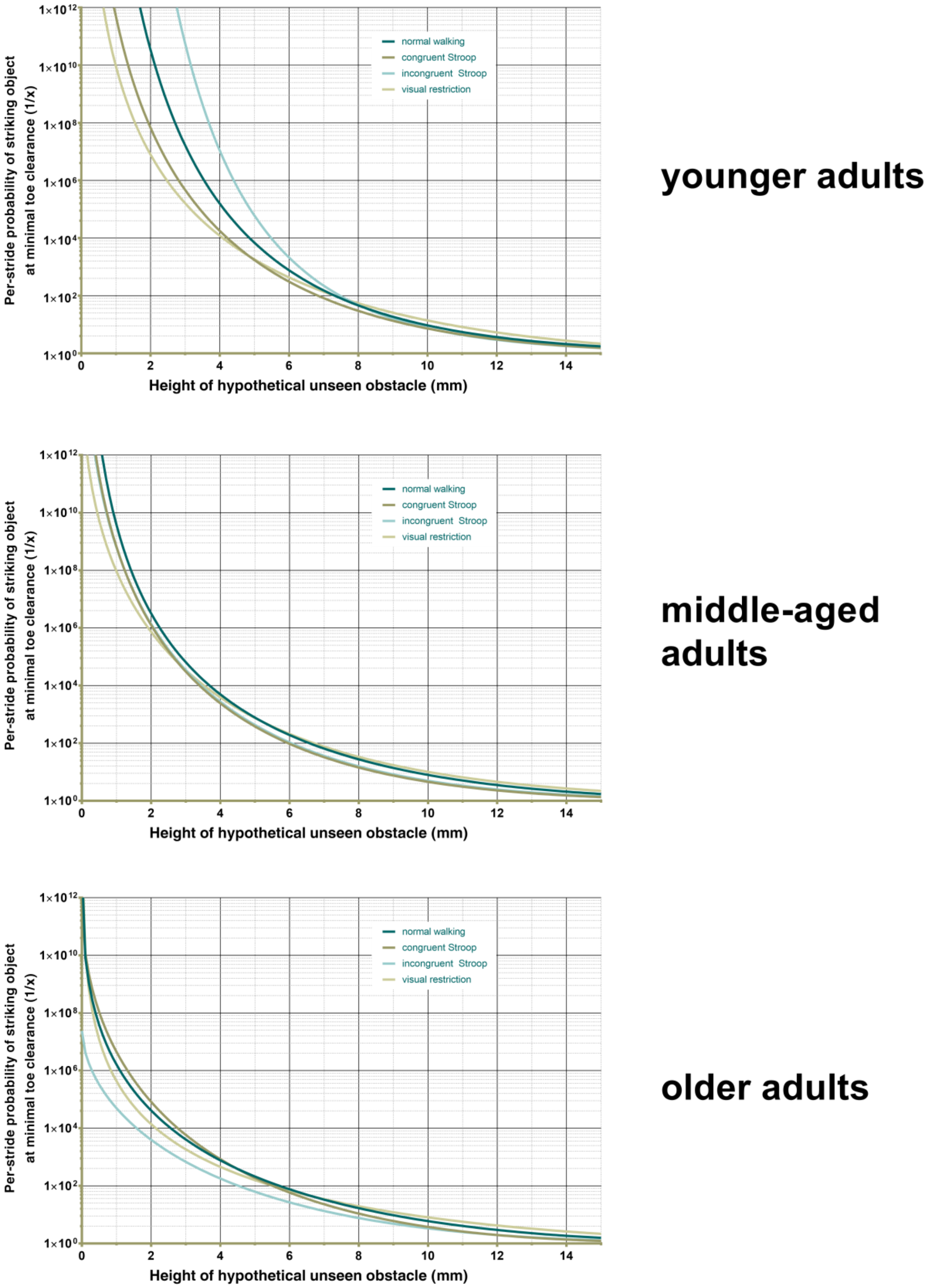

Supplementary Figure 3. Tripping probability modelling for (upper) healthy adults aged 20-39 years, (middle) 40-59 years and (lower) 60-80 years under different locomotor conditions. Modelling was based on the group frequency distributions and followed the approach taken by Best & Begg.[8]

Briefly, per-stride probabilities of striking a hypothetical, unseen obstacle of a given height at MTC are modelled based on MTC frequency distributions, including skewness and kurtosis. Data for one individual was removed from the younger age group for the calculation as inclusion of this data caused the probability modelling for the incongruent Stroop task to fail.

Supplementary Table 1.

	Additional task	Step length		Step width		C7 marker trajectory length	C7 marker mediolateral deviation
		Mean (mm)	Variability (CoV; %)	Mean (mm)	Variability (CoV; %)	Mean (mm)	Mean (mm)
Young adults (20 – 39 years)	None (baseline)	582.8	1.9	71.8	30.7	176.3	43.5
	Congruent	583.7	1.9	76.1	30.1	189.3	49.2
	Incongruent	579.4	1.9	77.8	26.2	186.6	49.3
	Visual restriction	575.8	2.4	72.2	31.7	169.9	*42.2*
Middle aged adults (40 – 59 years)	None (baseline)	563.5	2.0	78.3	25.7	171.7	44.2
	Congruent	572.1	1.8	82.2	24.8	188.1	50.9
	Incongruent	565.8	2.0	86.6	22.8	187.3	**52.0**
	Visual restriction	566.4	2.6	78.9	28.9	172.5	*43.3*
Older adults (60 – 80 years)	None (baseline)	531.0	2.5	71.1	30.9	164.0	45.6
	Congruent	541.9	2.5	71.7	31.1	**186.7**	**52.7**
	Incongruent	536.6	3.0	76.7	33.9	**190.2**	**55.5**
	Visual restriction	524.6	3.7	72.4	34.2	*168.0*	*45.4*

Supplementary table. Gait parameters under four walking conditions on the treadmill; normal walking (no additional cognitive load), walking while performing a congruent Stroop task, walking while performing an incongruent Stroop task and walking with vision restricted. Step length is the distance in the progression axis from heel strike to ipsilateral heel strike. Step width is the distance in the lateral axis from heel strike to contralateral heel strike. Bold type indicates significant change to baseline walking condition, italic type indicates significant change between visual restriction and both Stroop task conditions ($p \leq 0.05$; linear mixed model with post-hoc pairwise comparisons and Bonferroni correction). C7; marker on 7th cervical spinous process, CoV; coefficient of variation.
